# Acute L-Leucine Supplementation and Sprint Exercise Elicit Distinct Appetite and Inflammatory Responses in Persons with Overweight: A Randomized, Counterbalanced, and Crossover Design Study

**DOI:** 10.3390/nu18040614

**Published:** 2026-02-13

**Authors:** Elias de França, Ronaldo Vagner Thomatieli-Santos, Rita de Cássia Aquino, Mykaelen Malaquias Cavalcante, Beatriz Rugila Salvalágio, Peter Hofmann, Raul A. Martins, Liliana C. Baptista, Fabio Santos Lira, Erico das Chagas Caperuto

**Affiliations:** 1 Interdisciplinary Graduate Program in Health Sciences, Universidade Federal de São Paulo, São Paulo 04024-001, SP, Brazil; ronaldo.thomatieli@unifesp.br; 2 Laboratory of Human Movement, São Judas Tadeu University, São Paulo 05503-001, SP, Brazil; rcaquino@uol.com.br (R.d.C.A.); mykanutricionista@gmail.com (M.M.C.); ericocaperuto@gmail.com (E.d.C.C.); 3 Graduate Program in Psychobiology, Universidade Federal de São Paulo, São Paulo 04021-001, SP, Brazil; beatriz.rugila@unifesp.br; 4 Exercise Physiology, Training & Training Therapy Research Group, Department of Human Movement Sciences, Sport and Health, University of Graz, 8010 Graz, Austria; peter.hofmann@uni-graz.at; 5 Research Unit for Sport and Physical Activity, Faculty of Sport Sciences and Physical Education, University of Coimbra, 3000 Coimbra, Portugal; raulmartins@fcdef.uc.pt (R.A.M.); lbaptista@fcdef.uc.pt (L.C.B.); 6 Post-Graduation Program in Movement Sciences, Exercise and Immunometabolism Research Group, Department of Physical Education, State University of São Paulo (UNESP), Presidente Prudente 01049-010, SP, Brazil; fabioslira@gmail.com; 7 CIPER, Faculty of Sport Sciences and Physical Education, University of Coimbra, 3000 Coimbra, Portugal

**Keywords:** appetite control, L-leucine, exercise, overweight

## Abstract

Objectives: Our objective was to evaluate the acute effect of L-leucine supplementation and high-intensity sprint exercise on appetite-controlling neuropeptides and their association with the subjective perception of appetite (SPA), satiety (SPS), food intake, and inflammatory response in overweight participants. Methods: In a double-masked, randomized, counterbalanced, and crossover design, 12 sedentary overweight adult men performed four experiments: (1) exercise and L-leucine (EX-Leu), (2) exercise and placebo (EX-PLA), (3) L-leucine without exercise (SED-Leu), and (4) placebo without exercise (SED-PLA). The supplementation consisted of three daily doses of 70 mg/kg body weight of L-leucine or placebo (on the day of exercise and one day after). During the experiments, we recorded the food intake, SPA, and SPS, and evaluated the neuropeptides (GLP-1, PYY, CCK, and ghrelin) and cytokines (IL1-beta, IL-6, IL-10, and TNF-α) in peripheral blood. The acute exercise trial consisted of four sets of 30 sec cycle ergometer sprint exercises. Results: EX-Leu, EX-PLA, and SED-Leu decreased SPA, compared to SED-PLA; only EX-PLA improved SPS; EX-PLA and EX-Leu reduced food intake. GLP-1 decreased in the EX-PLA trial compared to SED-Leu. IL-6 and IL1-β levels increased in the EX-Leu trial compared to SED-PLA. An anti-inflammatory profile was identified in the EX-PLA trial compared to the other trials. Both neuropeptides (increased) and cytokines (a pro-inflammatory profile) were associated with changes in SPA, SPS, and food intake. Conclusions: The acute inflammatory balance induced by EX-Leu seems to improve appetite control. Sprint exercise had a consistent acute anorexic effect, while isolated L-leucine decreased SPA, but their impact on SPS and food intake is not clear (FAPESP grants: 2020/09936-2 and 2021/03601-1).

## 1. Introduction

The dysregulation in the satiety mechanism in the hypothalamus in obese animal models is triggered by a chronic systemic low-grade inflammatory process induced by a high-carb/high-fat diet. A high-carb/high-fat diet increases plasma concentrations of lipopolysaccharides (LPS), tumor necrosis factor alpha (TNF-α), interleukins 1 beta (IL1-β), and IL-6, causing a chronic hyperphagia [1,2,3], insulin resistance, type 2 diabetes, and associated comorbidities [4]. On the other hand, nutritional intervention might always be adopted as a strategy to intervene in these changes [4]. L-leucine supplementation has been shown to be effective in inducing increased plasma levels of anorectic peptides [5,6], with an anorexic effect in animal models by inducing activation in neuron pathways in regions of the central nervous system that are responsible for appetite inhibition [7,8]. In rats [9,10], a central infusion of L-leucine into the paraventricular area induces an anorectic effect. However, to induce an anorectic effect, the animal studies [10,11] have identified that oral intervention requires a bolus of 3 g/kg of body weight of protein for L-leucine to significantly reach the paraventricular area. In humans, this modality of protein intake is impractical. Therefore, isolated supplementation is necessary to test its effect on appetite inhibition.

To verify if L-leucine inhibits appetite, we recently tested three daily oral doses of 70 mg/kg of body weight in lean and well-trained individuals. We identified that supplementation with L-leucine mitigated the orexigenic effect induced by physical exercise [12]. Nevertheless, these data cannot be extrapolated for persons with overweight/obesity since it was identified that they apparently do not respond to appetite reduction under sedentary conditions when supplemented with high doses of L-leucine [13]. On the other hand, the same study identified that eutrophic individuals had a significant reduction in appetite, suggesting that persons with overweight/obesity may not respond to L-leucine supplementation, apparently due to insulin resistance. Thus, strategies to overcome insulin resistance are necessary. We hypothesize that physical exercise, a well-known powerful insulin mimicker, could be used as a strategy to overcome insulin resistance [14]. In this sense, it is likely that L-leucine supplementation, when combined with physical exercise, may have a significant effect on reducing appetite in persons with overweight. To our knowledge, no studies have verified in persons with overweight/obesity the efficacy of combining L-leucine supplementation with physical exercise on the appetite control response.

Beyond an insulin mimicker [14], evidence in animal models suggests that the restoration or maintenance of satiety mechanisms may occur with physical exercise practice, since acute physical exercise induces an increase in anorectic neuropeptides such as GLP-1 and CCK while decreasing ghrelin [15,16]. Furthermore, in an animal model, it is well documented that physical exercise induces an anti-inflammatory effect through the induction of acute inflammation in each exercise session, inducing an increase in IL-1-β and IL-6 and, consequently, a counter-regulatory effect via IL-10, which has a direct implication in improving insulin and leptin sensitivity and appetite control [17]. However, the relationship between the inflammatory/anti-inflammatory/appetite balance in physical training has not yet been tested in humans. In humans, some studies suggest that the anorectic response to exercise is mediated by neuropeptides and is dependent on intensity and plasma IL-6 levels [15,16]. Meanwhile, data from our laboratory [12] and others [18] suggest that well-trained individuals exhibit an orexigenic response to exercise, regardless of intensity. From animal model data [1], it has been hypothesized that the anorexigenic effect of exercise appears to occur in rats with previously established chronic inflammation induced by a hypercaloric diet rich in fat or carbohydrates, but not in lean rats with a regular diet using standard chow. Therefore, the impact of the inflammatory balance induced by physical exercise on the satiety/appetite mechanism in humans remains to be established.

Therefore, this study aimed to evaluate in overweight adult men the acute effect of L-leucine supplementation and physical exercise on the inflammatory response, appetite-controlling neuropeptides, and their impacts on the perception of satiety, appetite, and caloric intake. Our primary hypothesis is that physical exercise or L-leucine supplementation improves satiety and suppresses appetite and food intake. Our second hypothesis is that there is a significant association between satiety, appetite, food intake, and the balance of pro- and anti-inflammatory profiles, as well as neuropeptides related to appetite control.

## 2. Materials and Methods

### 2.1. Study Design

This was a double-blind, randomized, counterbalanced, crossover study. Participants accomplished four experiments:

(1) Sedentary lifestyle plus placebo supplementation (SED-PLA);
(2) Sedentary lifestyle plus L-leucine supplementation (SED-Leu);
(3) High-intensity sprint interval exercise plus placebo supplementation (EX-PLA);
(4) High-intensity sprint interval exercise plus L-leucine supplementation (EX-Leu).

**Figure 1:**
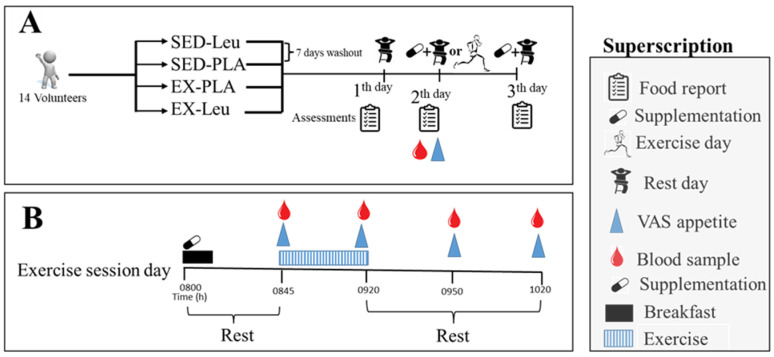
Experimental design of L-leucine supplementation and appetite assessment in high-intensity sprint interval exercise. (**A**), general design. (**B**), second day of experiment with exercise session intervention. Acronym: EX, exercise; Leu, Leucine; PLA, placebo; VAS, visual analog scale for appetite assessment.

As shown in Figure 1A, each experiment was conducted over a three-day period: on day 1, participants began recording their dietary intake over a 24 h period. These data were then used to estimate their caloric intake at baseline. On day 2, participants were supplemented with L-leucine or placebo and performed a high-intensity sprint exercise session (or remained sedentary). They also recorded their caloric intake on day 2, as well as their appetite perception before and after the physical exercise, along with blood samples (more details about the procedures are shown in Figure 1B and below). On day 3, participants continued to be supplemented with L-leucine or placebo and recorded their caloric intake. In the sedentary protocol, volunteers performed all procedures as in the protocols for the day with physical exercise but without physical exercise, in which participants remained seated in the laboratory to collect all the same variables collected during exercise. During this time, volunteers were allowed to watch TV and use their cell phones or computers.

As illustrated in Figure 1B (second day of the experiment and exercise session), volunteers arrived at 8:00 a.m. (fasting) at the Human Movement Laboratory and ingested the first dose of the supplement followed by a standardized snack (55% carbohydrates; 30% fat; 15% protein) providing 7 kcal per kilogram of body weight. Volunteers were asked to eat the snack within a 15 min period. The same snack was given in all four experiments, so there are no differences in caloric intake or nutrient composition. This breakfast was counted in the total caloric intake on the second day of the experiment. The exercise session was performed 30 min after the snack.

To exclude the carryover effect, after performing an experiment, volunteers had a 7- to 14-day washout interval before participating the next experiment (general study design shown in Figure 1A). This period of time is enough to clear possible physiologic effects of acute high sprint interval exercise [19] and L-leucine supplementation [20]. Also, to exclude the carryover effect, we performed randomization and counterbalancing of the experiments: the sequence of collections for each participant was made on the website http://www.jerrydallal.com/random/permute.htm (accessed on 2 August 2021), a website that performed randomization of the collections (using balanced permutation with 14 volunteers for four treatments).

### 2.2. Participant Characteristics

Participants were sedentary, overweight, and healthy adult males (age, 30 ± 10 years; height, 174 ± 8 cm; body weight, 83 ± 21 kg) considered fit to exercise. Participants presented a medical certificate assuring the absence of cardiovascular, muscular, joint, or bone problems that could put them at risk of clinically significant harm while participating in the study. Individuals with a body fat percentage range of >15% <30% (identified by bioimpedance), associated with a BMI of >25 <35, were included in the final sample of this study.

The sample size calculation was made using PS software (Version 3.1.2 Dupont and Plumer) based on the variation in appetite perception 30 min after exercise (1.7 ± 2.0 cm) described by Islam et al. [15], and the decrease in caloric intake after acute BCAA supplementation by Salcido et al. [13] with alpha (type I error) at 0.05 and beta (type II error) at 0.80, which estimated that the total inclusion of, respectively, 13 and 14 participants (in the crossover model) would be sufficient. We started with 14 participants, but eventually a different participant number will appear in the results. The number of participants is not the same in all results because some technical aspects interfered with some samples. The raw data will show the same sample number we described here.

Individuals who smoked or used any sports supplement (such as caffeine in capsules) or followed a diet prescribed by a nutritionist were excluded from the sample. Individuals who used medications/drugs that alter appetite were also excluded from the study. Participants were not asked to abstain from any type of food (such as coffee) to not disrupt their dietary pattern, but were asked to avoid alcoholic beverages on the days of the experiments.

Volunteers were instructed to not engage in other types of moderate to vigorous physical activity on the three days of the experiment.

The data were collected after volunteers had been briefed and familiarized with the study procedures and signed the informed consent form for the project, which was approved by the ethics committee of the Federal University of São Paulo and São Judas Tadeu University (No. 4.846.400).

### 2.3. Supplementation Protocol

In the four experiments, the volunteers ingested two types of supplementations: the first with L-leucine + isomaltulose supplementation followed by physical exercise (EX-Leu) or sedentary lifestyle (SED-Leu) and the second with placebo supplementation (isomaltulose) followed by physical exercise (EX-PLA) or sedentary lifestyle (SED-PLA)—Figure 1A.

As illustrated in Figure 1A, there were two days of supplementation. L-leucine dosage (Guki Alimentos LTDA, São Paulo, Brazil) consisted of three doses (morning, afternoon, and evening, every 8 h) of 70 mg/kg of body weight per dose, i.e., approximately 5 g of L-leucine for a 70 kg person (a dose sufficient to reach plasma concentration related to the anorexic effect in animals, as verified by Blouet et al. [9]). The supplement was administered in powder form and diluted in 200 mL of water. To mask the bitter taste of L-leucine, it was mixed with 70 mg/kg of body weight of lemon-flavored isomaltulose. The first dose was administered in a fasted state, followed by breakfast; the second dose was given in the afternoon (8 h after the first dose), and the third dose in the evening (8 h after the second dose).

Placebo supplementation consists of doses of 140 mg/kg of body weight of lemon-flavored isomaltulose, following the same pattern of L-leucine. Isomaltulose was chosen because of its medium glycemic index, which has less impact on the glycemic response of participants.

### 2.4. Exercise Session

The exercise session occurred 30 min after supplementation and breakfast. Before the exercise, the volunteers performed a 10 min warm-up at 70% of maximum heart rate (HRmax). The main exercise session consisted of 4 sets of 30 s of maximum effort cycle ergometer sprint interval training (SIT) against a resistance of 0.075 kg/kg of body weight. Active rest between sets was 4.5 min at 50 rpm, at an intensity of 30 W. A passive rest was not used in this study because, in the pilot study, some individuals experienced severe blood pressure oscillation with symptoms of nausea and vomiting during such a passive rest period. The sprint exercise sessions were monitored with a gas analyzer (VO2000 portable gas analyzer, Imbramed, Porto Alegre, Brazil) to quantify oxygen consumption (VO_2_) and carbon dioxide production (VCO_2_), as well as heart rate monitoring (Polar T31, Kempele, Finland).

### 2.5. Data Collection

#### 2.5.1. Food Intake Assessment

To assess food intake (kcal), volunteers were instructed to fill out a food diary to quantify and characterize the food consumed over three days, including the type of food and method of preparation. Recording was performed on day one before supplementation and on the first and second days of supplementation (Figure 1A). Participants were instructed by an experienced nutritionist on how to complete the diaries.

To assess food consumption, food diaries were provided to all participants on the designated days. The diaries involved recording and quantifying all foods and beverages consumed, from breakfast to supper, along with details on the characteristics of the food, preparation methods, and portion sizes. Participants were instructed not to alter their usual food intake. An experienced nutritionist provided guidance on how to complete the diary and also instructed participants on portion sizes. A photo tutorial was provided with the food diary to guide participants on food portions. After collection, the records were reviewed by a nutritionist, who converted the quantities of food and preparations listed in the diaries from household measurements into grams or kcal. A standardization of measurements was developed based on the ‘Table for Evaluating Food Consumption in Household Measurements’ [21] and the ‘Manual of Recipes and Household Measurements for Calculating Food Surveys’ [22].

The calculation of food and ingredient intake in kcal was performed using the Nutrition Data System for Research (NDSR) Software, version 2013, from the University of Minnesota. The program is considered a valid tool for calculating food intake, based on the table developed by the United States Department of Agriculture (USDA).

#### 2.5.2. Subjective Perception of Appetite and Satiety

To assess the subjective perception of appetite (SPA) and satiety (SPS), a questionnaire was used with items evaluating appetite and satisfaction associated with a visual analog scale. This questionnaire was previously validated in English [23] and was recently translated into Portuguese, where its reproducibility was tested, yielding an intraclass correlation of 0.98 [24].

The questionnaire assessed three aspects: (1) desire to eat (hunger), (2) desire for certain types of food (sweet, salty, fatty, and tasty, i.e., appetite), and (3) satiety. The desire to eat and satiety were measured on a 100-point scale (0 = none and 100 = strong desire). To do this, participants were asked to draw a vertical line on the scale to describe, from 0 to 100, the magnitude of their desire to eat a food or their satiety level. Subsequently, for statistical analysis, the values were quantified using a ruler to determine the hunger, appetite, and satiety scores.

The questionnaire was administered on the day of the sprint exercise session, in a fasted state (45 min before exercise) and immediately before exercise (which happened 30 min after the snack and supplementation), as well as immediately after, and 30 and 60 min after the exercise session (Figure 1B).

To compare subjective appetite perception (SPA) between conditions, the average of the five questions assessing desire to eat was calculated (hunger and appetite).

#### 2.5.3. Blood Sample Collection and Treatment

A total of 60 mL (15 mL per collection) of venous blood (in EDTA tubes) was drawn from the median cubital vein to assess peptides related to appetite (total GLP-1, acylated ghrelin, PYY, and CCK), as well as cytokines (IL-1β, IL-6, TNF-α, and IL-10). The blood samples were collected before exercise, immediately after exercise, and 30 and 60 min post-exercise (see Figure 1B).

To avoid protein degradation in the peptides assessment, 100 μL of Phenylmethylsulfonylfluoride (PMSF) was added for each mL of total blood sample (to reach a concentration of 17.4 mg/mL). All tubes were inverted 10 times and centrifuged for 10 min at 3000× *g* to separate the plasma. PMSF was previously suspended in 100% alcohol and kept in the refrigerator under an aluminum foil wrapping to avoid light interaction.

In a different vial for cytokine assessment, 10 μL of LPS (*Escherichia coli*, type 0111: B4; Sigma, St. Louis, MO, USA) was added for each mL of total blood sample (to reach a concentration of 10 ng/mL). The vial was incubated for 60 min with constant and slow rotation. Then, the blood sample was centrifuged for 10 min at 3000× *g* to separate the plasma. LPS was suspended in a distilled water solution and kept in the refrigerator.

Blood plasma was stored at −80 °C for later analysis with commercial Enzyme-Linked Immunosorbent Assay Kits (Elabscience Biotechnology Inc., Houston, TX, USA).

### 2.6. Statistical Analysis

Data are presented as a mean and confidence interval (CI) 95% or standard deviation (SD). To identify differences between treatments, we used the mixed linear model followed by the LSD test post hoc. As fixed effects, we entered treatment conditions (SED-Pla, SED-Leu, EX-Pla, and EX-Leu) and time-points of assessment. As the data were from the same volunteers and were used several times, the participant IDs were used as random effects (intercept model). To calculate the area under the curve, the mixed linear model was applied, using the treatment conditions (SED-Pla, SED-Leu, EX-Pla, and EX-Leu) as fixed effects and the volunteers (intercept model) as random effects. To compare inflammatory balance (IL-10//IL-6, IL-10//IL1-β, and IL-10//TNF-α ratio) across treatments, the data were standardized to z-score. The delta in appetite and satiety perception was calculated as the fasting state value (baseline) minus the respective values in the time-point assessments. An exploratory decision tree regression (DTR) model was performed (using the CHAID spontaneous growth method) to identify the most influential variables that could explain the variation in food intake, appetite, and satiety perception. As a dependent variable, we used food intake and the delta of the perception of appetite and satiety. Cytokines and neuropeptides were used as independent variables. The sequence of the four experiments was used as a covariate. The parents’ nodes (leaves) of the DTR were pruned to compare at least 30 samples in each node, and the child nodes were pruned to compare 20 samples. We validated all DTR tests with K-fold cross-validation (10-fold). All analyses were performed using the IBM SPSS Statistics for Windows (version 27.0, IBM Corp, Armonk, NY, USA). Significance was set at *p* ≤ 0.05.

## 3. Results

### 3.1. Participant Description and Exercise Characteristics

Table 1 presents the characteristics of participants in the fasting state before the start of the intervention, as well as the participant’s VO_2peak_. There were no significant statistical differences among the experimental groups in all satiety or hunger outcomes, and, on average, participants presented a low cardiorespiratory fitness.

**Figure 2:**
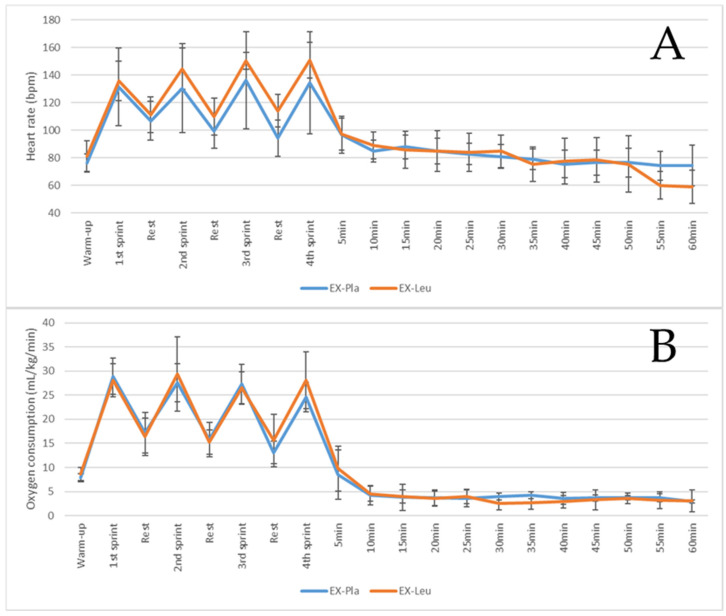
Heart rate response (**A**) and oxygen consumption (**B**) during a sprint interval training. Sprint data is the 30 s average of the highest values. Warm-up data are the last 30 s before the sprint. Post-exercise data are the 5 min average. Data are mean and standard deviation. EX-Pla, sprint interval training associated placebo supplementation; EX-Leu, sprint interval training associated with leucine supplementation.

Figure 2 illustrates, respectively, the heart rate and oxygen consumption response in two sessions of physical exercise. There was no statistical difference between all moments assessed for both heart rate and oxygen consumption. Therefore, we demonstrated that the two sessions of physical exercise were similar in inducing internal load response for both treatments.

### 3.2. Food Intake and Subjective Perception of Appetite and Satiety

**Figure 3:**
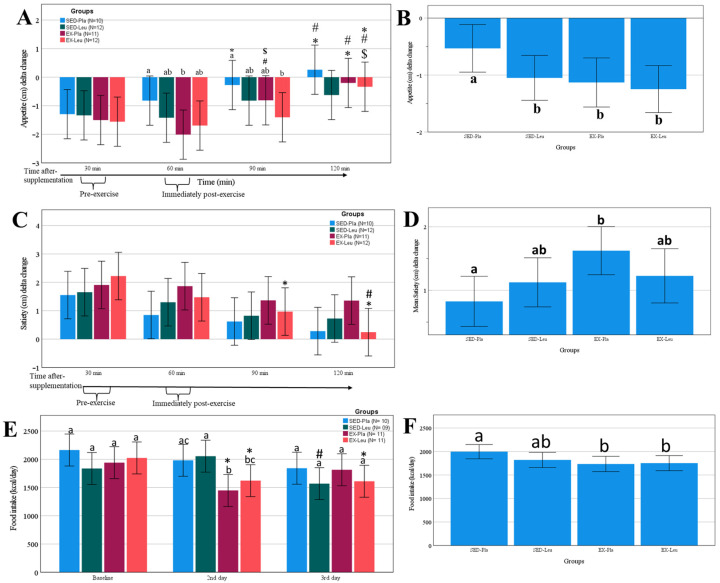
Change in appetite, satiety, and food intake after L-leucine supplementation or sprint interval training (exercise). (**A**), delta change in appetite in several moments; (**B**), area under the curve on appetite perception; (**C**), delta change in satiety in several moments; (**D**), area under the curve on satiety perception; (**E**), change in food intake in several moments; (**F**), area under the curve on food intake. SED-PLA, placebo supplementation without exercise; SED-Leu, L-leucine supplementation without exercise; EX-Pla, placebo supplementation associated with exercise; EX-Leu, L-leucine supplementation associated with exercise. Data are predicted values from a mixed linear model and 95% CI. In (**A**): *, *p* < 0.05 when compared to 30 min; #, *p* < 0.05 when compared to 60 min; $, *p* < 0.05 when compared to 90 min. In (**A**), different letters denote statistical differences between treatments when compared to the same time-point. In (**B**), different letters denote statistical differences between treatments. In (**C**): *, *p* < 0.05 when compared to 30 min; #, *p* < 0.05 when compared to 60 min. In (**D**), different letters denote statistical differences between treatments. In (**E**): *, *p* < 0.05 when compared to baseline; #, *p* < 0.05 when compared to 2nd day. In (**E**), different letters denote statistical differences between treatments when compared at the same time-point. In (**F**), different letters denote statistical differences between treatments.

The subjective perception of appetite decreased after the meal in all treatments (Figure 3A). The sedentary placebo condition (SED-Pla) brought back appetite perception more quickly. The area under the curve demonstrated that the three treatments (i.e., SED-Leu, EX-Pla, and EX-Leu) were effective in suppressing appetite compared to the control condition (Figure 3B).

The satiety perception increased in all treatments after the meal (Figure 3C). We noticed a difference between the treatments when we analyzed the area under the curve, which shows that the EX-Pla had a higher perception of satiety when compared to the SED-Pla control condition (Figure 3D).

Both post-exercise moments with sprint exercise (EX-Pla and EX-Leu) showed a decrease in caloric intake (Figure 3E). One day after the sprint exercise, the caloric intake (Figure 3E) was suppressed in conditions with leucine supplementation. The area under the curve shows that both treatments with sprint exercise supplementation suppressed caloric intake (Figure 3F).

### 3.3. Neuropeptides Related to Appetite and Satiety Response to L-Leucine Supplementation and Exercise

**Figure 4:**
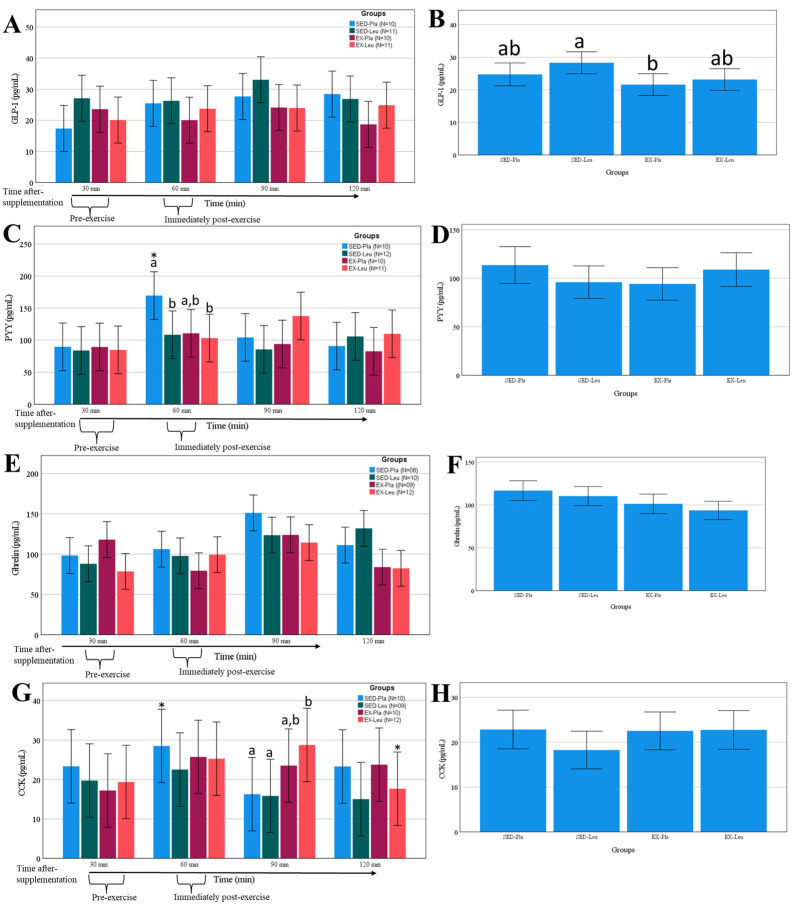
Change in glucagon-like peptide-1 (GLP-1), cholecystokinin (CCK), peptide YY (PYY), and ghrelin after L-leucine supplementation or sprint interval training (exercise). (**A**), change in GLP-1 in several moments; (**B**), area under the curve on GLP-1; (**C**), change in PYY in several moments; (**D**), area under the curve on PYY; (**E**), change in ghrelin in several moments; (**F**), area under the curve on ghrelin; (**G**), change in CCK in several moments; (**H**), area under the curve on CCK. SED-Pla, placebo supplementation without exercise; SED-Leu, L-leucine supplementation without exercise; EX-Pla, placebo supplementation associated with exercise; EX-Leu, L-leucine supplementation associated with exercise. Data are predicted values from a mixed linear model and 95% CI. In (**B**), different letters denote statistical differences between treatments. In (**C**) *, *p* < 0.05 when compared to 90 min time-point. In (**C**), different letters denote statistical differences between treatments when compared at the same time-point. (**G**): *, *p* < 0.05 when compared to 90 min time-point. In (**G**), different letters denote statistical differences between treatments when compared at the same time-point.

There was no clear effect of exercise or L-leucine supplementation on GLP-1, PYY, or ghrelin concentrations (Figure 4A, B, C, D, E, and F, respectively). On the other hand, the CCK concentration increased post-exercise for both treatments (Figure 4G,H).

### 3.4. Cytokines Response to L-Leucine Supplementation and Exercise

**Figure 5:**
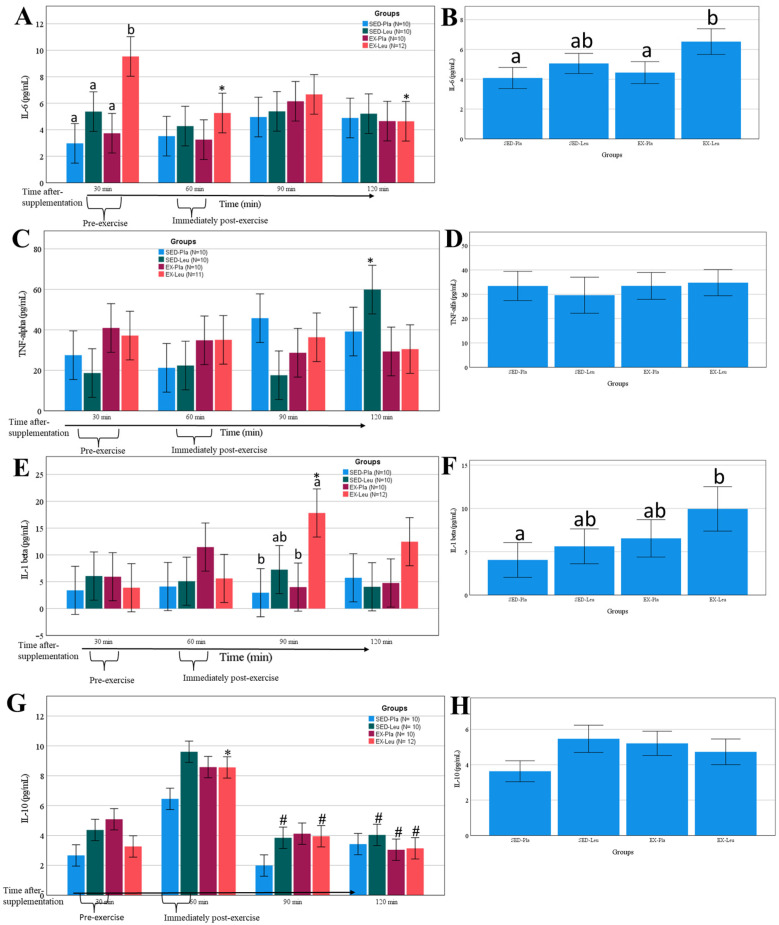
Change in interleukin-6 (IL-6), tumor necrosis factor alpha (TNF-α), interleukin-1 beta (IL-1 β), and interleukin-10 (IL-10) after L-leucine supplementation or sprint interval training (exercise). (**A**), change in IL-6 in several moments; (**B**), area under the curve on IL-6; (**C**), change in TNF-α in several moments; (**D**), area under the curve on TNF-α; (**E**), change in IL-1β in several moments; (**F**), area under the curve on IL-1β; (**G**), change in IL-10 in several moments; (**H**), area under the curve on IL-10. SED-Pla, placebo supplementation without exercise; SED-Leu, L-leucine supplementation without exercise; EX-Pla, placebo supplementation associated with exercise; EX-Leu, L-leucine supplementation associated with exercise. Data are predicted values from a mixed linear model and 95% CI. In (**A**): *, *p* < 0.05 when compared to EX-Leu 30 min time-point. In (**A**), different letters denote statistical differences between treatments when compared at the same time-point. In (**B**), different letters denote statistical differences between treatments. In (**C**): *, *p* < 0.05 when compared to other SED-Leu time-points. In (**E**) *, *p* < 0.05 when compared to other EX-Leu 30 and 60 min time-points. In (**E**), different letters denote statistical differences between treatments when compared at the same time-point. In (**F**), different letters denote statistical differences between treatments. In (**G**) *, *p* ≤ 0.09 when compared to 60 min time-point; #, *p* ≤ 0.10 when compared to 60 min time-point.

The L-leucine supplementation increased IL-6 (Figure 5A,B) and TNF-α (Figure 5C) concentrations. The IL-1β increased in the EX-Leu treatment (Figure 5E,F). A trend to increase occurred in IL-10 immediately post-exercise in the L-leucine and exercise treatment (EX-Leu) (Figure 5G).

**Figure 6:**
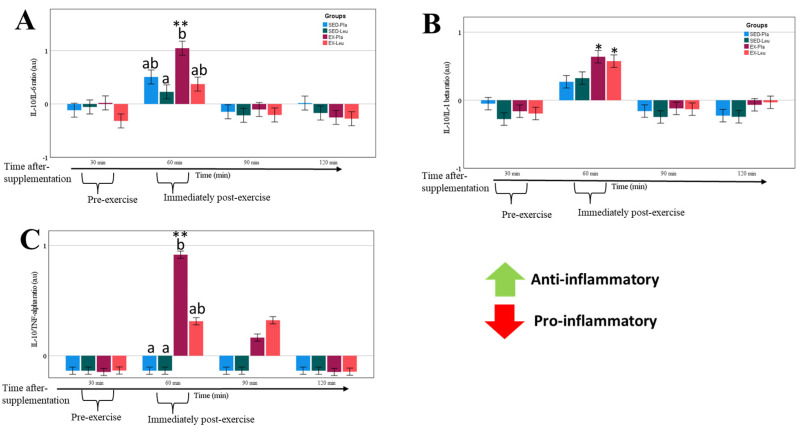
Predicted values (delta change Z-score and 95% CI) in several moments of the inflammatory balance after breakfast in four treatments: (1) placebo supplementation without exercise (SED-Pla); (2) L-leucine supplementation without exercise (SED-Leu); (3) placebo supplementation associated with exercise (EX-Pla); and L-leucine supplementation associated with exercise (EX-Leu). (**A**), IL-10//IL-6 balance. (**B**), IL-10//IL1-β balance. (**C**), IL-10//TNF-α balance. *, *p* < 0.05 when compared to 30 min time-point. **, *p* < 0.05 when compared to all other EX-Pla time-points. In (**A**,**C**), different letters denote statistical differences between treatments when compared at the same time-point.

Sprint exercise (only in EX-PLA) induced an anti-inflammatory balance (Figure 6A–C), which is apparently mitigated when sprint exercise was combined with the L-Leucine supplementation (i.e., EX-Leu).

### 3.5. The Interaction Between Cytokines and Neuropeptides Related to Appetite Control on Food Intake, Subjective Perception of Appetite and Satiety

**Figure 7:**
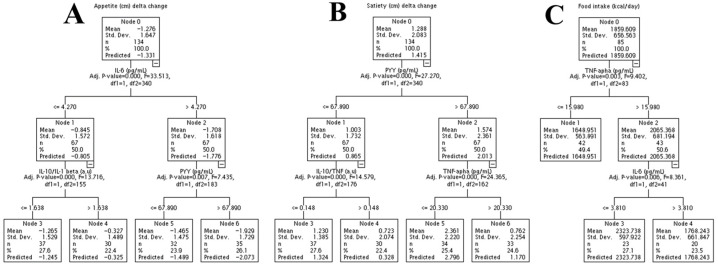
Decision tree regression of the change (delta) of appetite perception (**A**), satiety (**B**), and food intake (**C**), with markers related to pro- or anti-inflammatory cytokines and hormones/peptides related to appetite control. IL-6, interleukin-6; IL-1 beta, interleukin-1 beta; IL-10, interleukin-10; PYY, peptide YY; TNF-α, tumor necrosis factor alpha. >, above; <=, less or equal.

With the intention of investigating if the pro- or anti-inflammatory response of cytokines and the peptide hormones related to appetite control can explain the variation in the subjective perception of appetite, satiety, and food ingestion, we used a machine learning approach, particularly the decision tree regression technique (Figure 7).

In Figure 7A, node 2, it is shown that an increase in IL-6 higher than 4 pg/mL is associated with a decrease in appetite perception. Instead, in node 1, when there is a lower concentration of IL-6 (≤4 pg/mL), the decrease in appetite perception is mitigated. Node 2 is branched into nodes 5 and 6, showing that an increase in PYY (>67.890 pg/mL) in response to treatments is associated with a greater decrease in appetite perception. Node 1 is divided into nodes 3 and 4. Nodes 3 and 4 demonstrate that individuals with an anti-inflammatory balance (>1.581 a.u.) of the IL-10/IL-1β ratio have a decrease in appetite perception blunted in response to treatments. Specifically, in node 3, individuals with a pro-inflammatory balance (≤1.6581 a.u) of the IL-10/IL-1β ratio demonstrate a more robust decrease in appetite in response to treatments. In contrast, in node 4, for individuals with an anti-inflammatory balance (≥1.6581 a.u) of the IL-10/IL-1β ratio, there is no change in appetite perception. This suggests that individuals with a high anti-inflammatory balance do not have a decrease in appetite in response to treatments (exercise or L-leucine supplementation). Together, these data suggest that an acute pro-inflammatory response related to IL-6, IL-10/IL-1β, and higher PYY is associated with a decrease in appetite perception.

The marker that best explains the variation in the subjective perception of satiety is PYY (Figure 7B). In Figure 7B, it is shown that concentrations above 67.89 pg/mL of PYY are associated with greater satiety when compared to a lower PYY concentration (≤67.89 pg/mL). Node 2 is divided into nodes 5 and 6, showing that, also, an increase in TNF-α in response to treatments seems to have blunted the effect of PYY on satiety. Node 1 branches into nodes 3 and 4, showing that low concentrations of PYY and a pro-inflammatory balance in node 3 are associated with an increase in satiety.

A TNF-α above 15 pg/mL was associated with a higher calorie intake (Figure 7C, node 2), while concentrations lower than 15 pg/mL were associated with a lower calorie intake (node 1). Node 2 is branched into nodes 3 and 4, suggesting that, in those individuals that have a higher plasma TNF-α concentration, an increased IL-6 mitigates the increase in caloric intake.

## 4. Discussion

The aim of this study was to evaluate in persons with overweight the acute effect of L-leucine supplementation and sprint exercise on inflammatory responses, appetite-controlling neuropeptides, and their impact on the perceptions of satiety, appetite, and caloric intake.

The main finding of this study was that, in overweight men, the short high-intensity sprint exercise combined or not with L-leucine supplementation was associated with a decrease in the subjective perception of appetite, improved satiety, and consequently a decreased caloric intake on the day of physical exercise. On the other hand, the isolated supplementation of L-leucine was also associated with a decrease in appetite perception. However, no significant association was identified for satiety or caloric intake. Another important finding was that the change in the subjective perception of appetite and satiety, as well as caloric intake, was significantly associated with inflammatory balance and neuropeptide concentration.

Our protocol identified a significant association between isolated L-leucine supplementation and reducing subjective appetite perception, although the results on the perception of satiety were not statistically different. However, when L-leucine supplementation was associated with exercise, no difference in satiety was observed. Our findings are consistent with the literature, which suggests that L-leucine supplementation is associated with appetite suppression [7,8].

It is important to note that appetite is associated with the desire to eat and the search for food. Satiety, on the other hand, is the decrease in the desire to eat food during the food intake process. These two processes (i.e., appetite and satiety) are regulated by distinct neural mechanisms [25], which require different assessment approaches to evaluate them properly. For instance, studies have shown that, in humans, the supplementation of L-leucine increases satiety [5] and decreases the size of the food bolus ingested [13]. Therefore, we understand that our methodology for assessing satiety could be improved.

It is important to remember that we combined L-leucine supplementation with sprint exercise to overcome the probable insulin resistance in persons with overweight, who may exhibit a blunted response to L-leucine for appetite control [13]. As we were unable to demonstrate an effect on satiety after the combination of L-leucine supplementation with exercise, this topic will require further investigation.

From a general perspective, our data show an acute anti-inflammatory response immediately after sprint exercise. Pro-inflammatory cytokines were particularly acutely elevated in the EX-Leu and the SED-Leu treatments, as indicated by the area under the curve. EX-PLA treatment showed an acute anti-inflammatory profile after exercise. However, when exercise was combined with L-leucine supplementation, this anti-inflammatory effect was blunted. Studies in animal models of cancer reported that chronic L-leucine supplementation increased the concentrations of several cytokines (TNF-α, interferon-gamma, IL-4, IL-6, and IL-10) in addition to increasing the TNF-α/IL-10 ratio [26], suggesting that L-leucine potentiates the phenotype. Also, studies with acute Leucine supplementation have identified an increased production of IL-15 and IL-5 in skeletal muscles and adipocytes of obese rats [27]. Furthermore, significant changes in gene expression in the immune system (signaling for an anti-inflammatory profile) have been identified in in vitro studies with equine cells [28]. In this sense, our data are in agreement with the literature, suggesting a significant impact of L-leucine supplementation on acute cytokine production.

Regarding IL-6, a significant change was observed only in the EX-Leu treatment, suggesting an interaction between sprint exercise and L-leucine supplementation in the acute modulation of plasma cytokine concertation. The area under the curve analysis confirms higher IL-6 concentrations in the EX-Leu treatment compared to both the SED-Pla and EX-Pla treatments. A recent study using skeletal muscle cell cultures with L-leucine at a concentration of 2.5 mM (an estimated concentration six times higher than what was used in our protocol [27]) from sedentary animals showed an increase in IL-6 concentration within the skeletal muscle in the absence of exercise [27]. Our sprint exercise protocol was unable to induce a significant increase in plasma IL-6 concentration in the EX-Pla condition despite the LPS blood stimulation technique. We hypothesized that sprint exercise would increase this cytokine, similarly to a previous study with the same exercise session and a comparable internal load response (heart rate and % of VO_2peak_) to the current study [15,16]. However, what we observed was a trend to increase in IL-10 at post-exercise, suggesting that our training protocol induced an anti-inflammatory shift instead of an expected pro-inflammatory one. In the long term, this anti-inflammatory response associated with exercise may be beneficial for improving appetite control [1]. Interestingly, the combination of sprint exercise with L-leucine supplementation resulted in higher plasma concentrations of IL-6, which mitigated the anti-inflammatory balance of exercise (as identified in EX-Pla). Future studies should, therefore, investigate whether the chronic L-leucine supplementation associated with sprint exercise has a positive repercussion in the inflammation/anti-inflammatory balance and appetite control.

It is important to note that neuropeptides related to appetite control were assessed after breakfast. We aimed to verify whether physical exercise or L-leucine supplementation has an additive effect with a snack (breakfast) on appetite and satiety perception. This approach was based on previous findings showing that carbohydrate consumption during breakfast increases GLP-1 and PYY concentration while decreasing ghrelin levels [29]. Therefore, strategies that enhance appetite suppression, reduce hunger, and increase satiety are essential for reducing excessive calorie intake. In this context, L-leucine supplementation did not induce significant changes in the analyzed hormones GLP-1 and ghrelin. On the other hand, the association of L-leucine with sprint exercise led to an increase in CCK 30 min after exercise. PYY concentration increased in response to the snack in placebo supplementation (SED-PLA treatment) but did not increase when individuals were supplemented with L-leucine or when they performed the sprint exercise. This suggests that the PYY response was attenuated in these treatments. Under fasting conditions, a previous study with a dose of 9.9 g (almost twice what we used in our study) identified an increase in CCK and a trend for a reduction in ghrelin when compared to the control group that had a saline solution but did not alter the concentrations of PYY or GLP-1 [30]. Thus, an additive effect of L-leucine supplementation with breakfast appears to occur in CCK but not in ghrelin and GLP-1. Furthermore, the fact that PYY does not increase in response to a breakfast associated with L-leucine supplementation should be further investigated, since our data show a significant association between PYY and satiety, as identified by the machine learning analysis.

Our machine learning approach, using decision tree regression, suggests that an acute inflammatory response (identified mainly by IL-6) is associated with a reduction in appetite perception and food intake. The reduction in appetite perception is intensified when PYY is increased. Additionally, increased PYY was significantly associated with improved satiety; however, this satiety perception is mitigated when TNF-α is increased. More interestingly, TNF-α seems to induce an increase in food intake, but this effect is mitigated by a higher IL-6 response to treatments. These data are consistent with the literature. It is well documented that, both acutely and chronically, a high-fat diet induces an inflammatory cascade (via TNF-α) in the hypothalamus, inducing the expression of neurons related to increased appetite [31]. Thus, it is plausible that, even if treatments increase PYY or IL-6 concentrations, the effects on satiety and food intake are smaller in the presence of high TNF-α concentrations (see Figure 7B,C).

Moreover, when IL-6 or PYY are at low concentrations, the acute inflammatory balance is associated with appetite suppression and improved satiety. This suggests that an acute pro-inflammatory balance induced by the treatments has the potential to decrease appetite and enhance satiety, as already demonstrated in an animal model [1]. It is important to emphasize that the perception of appetite, hunger, and satiety are three distinct concepts that manifest independently by different mechanisms [25] but become dysregulated in an obesogenic context via chronic low-grade inflammation (mainly via TNF-α) [2,3]. However, in an animal model, it is well described that moderate, continuous, and long-duration physical exercise induces acute inflammation (via IL-6) and consequently an anti-inflammatory counter-regulation through IL-10, which in the long term has a direct implication in the resolution of low-grade inflammation and consequently in the improvement of insulin–leptin sensitivity and appetite control [17]. Interestingly, our data demonstrate that the combination of L-leucine supplementation and sprint exercise has an acute effect on inducing a pro-inflammatory balance. Thus, future studies are needed to determine whether this combination has a long-term positive impact on appetite control. Finally, our data shed light on the literature related to the mechanisms of appetite control; that is, an acute inflammatory balance induced by sprint exercise associated with L-leucine supplementation in persons with overweight has the potential to induce better appetite control.

This study has some limitations. (1) In the statistical analysis, we chose to use the post hoc LSD test (which does not adjust for multiple comparisons), thus increasing the risk of a type 1 error. However, due to our small sample, the use of adjustments such as the Bonferroni test increases the risk of a type 2 error. Future studies can address the limitations of our study with a larger sample. (2) We chose to use a supplement diluted in water instead of capsules. In fact, even with the lemon flavor, L-leucine supplementation still has a slightly more bitter taste than the placebo. With this in mind, we recruited individuals who had not taken L-leucine powder diluted in water. In previous studies with L-leucine supplementation, the participants did not correctly identify L-leucine or placebo supplements [12]. In this study, participants were also uncertain whether they were taking the correct L-leucine or a placebo supplement. However, we did not perform statistical testing for this outcome in this study. Some strengths of this study should be noted. Our double-blind, crossover, randomized, and permutation-counterbalanced study design mitigates sample-related and data-collection noise.

This study has practical applications. Regular aerobic exercise, whether high-intensity or continuous and moderate, is associated with better appetite control, especially in overweight or obese individuals [18]. Furthermore, adding foods rich in L-leucine (protein) to the diet is also associated with improved satiety [5,6]. Therefore, individuals seeking to reduce caloric intake to combat obesity can adopt regular aerobic exercise and protein foods rich in L-leucine as part of their treatment.

## 5. Conclusions

We concluded that acute high-intensity sprint exercise was associated with an anorexic effect and an anti-inflammatory balance in persons with overweight.

Isolated L-leucine supplementation was associated with decreases in subjective appetite perception (SPA) but was not associated with a decrease in subjective satiety perception (SPS) or food intake. Also, acute L-leucine supplementation was associated with pro-inflammatory balance in persons with overweight.

Changes in neuropeptides and cytokines were associated with SPA, SPS, and food intake. Finally, an acute inflammatory balance induced by sprint exercise associated with L-leucine supplementation in persons with overweight has the potential to improve appetite control.

Studies with a large sample size need to be conducted to confirm these results. Also, long-term studies are needed to confirm whether the combination of these treatments is beneficial.

## Figures and Tables

**Table 1:** Participant description.

Variables	Treatment	N	Mean	SD	*p* ANOVA One-Way
VO_2peak_ (mL/kg/min)		12	36.7	3.7	
Glucose at fasting state (mg/mL)	SED-PLA	10	84.6	9.8	0.57
	SED-Leu	12	92.2	12.8	
	EX-PLA	11	86.5	13.1	
	EX-Leu	12	90.5	18.5	
Appetite in the fasting state (0 to 7 cm scale)	SED-PLA	10	2.7	1.6	0.91
	SED-Leu	12	2.6	1.6	
	EX-PLA	11	2.8	1.2	
	EX-Leu	12	2.3	1.6	
Satiety at fasting state (0 to 7 cm scale)	SED-PLA	10	2.1	1.6	0.64
	SED-Leu	12	2.2	1.8	
	EX-PLA	11	1.5	1.3	
	EX-Leu	12	1.9	1.3	
Hunger at fasting state (0 to 7 cm scale)	SED-PLA	10	4.8	1.8	0.87
	SED-Leu	12	4.3	2.0	
	EX-PLA	11	5.0	1.8	
	EX-Leu	12	4.6	2.6	

## Data Availability

The original data presented in the study are openly available in [Domus Dados: repositório de dados de pesquisa da Universidade Federal de São Paulo] [https://domusdados.unifesp.br/dataset.xhtml?persistentId=hdl:20.500.12682/rdp/MJXFD4] (accessed on 22 September 2025).

## Abbreviations

The following abbreviations are used in this manuscript:

CCK	Cholecystokinin
EX-Leu	High-intensity sprint interval exercise plus L-leucine supplementation
EX-PLA	High-intensity sprint interval exercise plus placebo supplementation
GLP-1	Glucagon-like peptide-1
IL	Interleukin
PYY	Peptide YY
SED-PLA	Sedentary lifestyle plus placebo supplementation
SED-Leu	Sedentary lifestyle plus L-leucine supplementation
SPA	Subjective perception of appetite
SPS	Subjective perception of satiety
TNF-α	Tumor necrosis factor alpha
